# Clinicopathological Implications of Maspin, CD8, and PD-L1 Expression in Liposarcomas

**DOI:** 10.3390/cimb47110935

**Published:** 2025-11-10

**Authors:** Andrei-Ionuț Patrichi, Ioan Jung, Simona Gurzu

**Affiliations:** 1 Department of Pathology, George Emil Palade University of Medicine, Pharmacy, Science and Technology, 540139 Targu-Mures, Romania; patrichi_andrei@yahoo.ro (A.-I.P.); jungjanos@studium.ro (I.J.); 2 Romanian Academy of Medical Sciences, 540139 Targu-Mures, Romania; 3 Research Center for Oncopathology and Translational Medicine (CCOMT), George Emil Palade University of Medicine, Pharmacy, Science and Technology, 540139 Targu-Mures, Romania

**Keywords:** liposarcoma, PD-L1 expression, CD8+ T lymphocytes, Maspin

## Abstract

Liposarcomas, the most common subtype of soft tissue sarcomas, show variable biological behavior and therapeutic response. Programmed death-ligand 1 (PD-L1) and cytotoxic T lymphocyte marker CD8 have been implicated in tumor immune evasion and prognosis in various malignancies, while Maspin, a tumor suppressor, has shown a negative prognostic impact in sarcomas. This study aimed to investigate the clinicopathological significance of PD-L1, CD8, and Maspin expression in liposarcomas. A retrospective analysis of 42 liposarcoma cases diagnosed between 2016 and 2023 was conducted. Immunohistochemical staining for PD-L1 (using DAKO 22C3 and 28-8 clones), CD8, and Maspin was performed. PD-L1 expression was assessed using the tumor proportion score (TPS) and tumor cell score (TC). CD8 expression was evaluated using an H-score, and Maspin positivity was assessed based on subcellular localization. Correlations with clinicopathological parameters were statistically analyzed using chi-squared and Fisher’s exact tests. Most liposarcomas exhibited low PD-L1 expression (<10%), but increased PD-L1 levels correlated with poor differentiation (G3), higher CD8 infiltration (H-score > 10%), and cytoplasmic Maspin positivity. Statistically significant associations were found between high PD-L1 expression and high CD8 infiltration (*p* = 0.007 for 22C3; *p* = 0.0331 for 28-8) and between PD-L1 positivity and Maspin expression (*p* = 0.003 for 22C3; *p* = 0.0113 for 28-8). CD8 infiltration was generally low across cases, and PD-L1 expression in inflammatory cells was noted predominantly in tumors with higher PD-L1 TPS/TC scores. High PD-L1 expression in liposarcomas is associated with poor tumor differentiation, increased CD8 infiltration, and Maspin positivity, suggesting an immune-evasive phenotype. Despite low overall expression rates, PD-L1 could serve as a prognostic biomarker and a potential target for immunotherapeutic strategies in liposarcomas. Further studies are necessary to standardize PD-L1 assessment and explore effective immunotherapy approaches for these tumors.

## 1. Introduction

Soft tissue tumors comprise a wide range of entities, amounting to 1% of malignant tumors with over 150 histological subtypes. Of these, liposarcomas represent the most prevalent subfamily of soft tissue sarcomas (STSs) with adipocytic origin, accounting for approximately 20% of all these tumors [1,2].

According to the latest edition of WHO Classification of Soft Tissue Tumors, these tumors are categorized into five subtypes: atypical lipomatous tumors (ALT)/well-differentiated liposarcoma (WDLPS), dedifferentiated liposarcoma (DDLPS), myxoid liposarcoma (MLPS), pleomorphic liposarcoma (PLPS), and myxoid pleomorphic liposarcoma (MPLPS) [3,4,5].

Standard treatment of localized liposarcomas, especially the well-differentiated ones, consists of surgical resection and chemotherapy. Nowadays there is a special focus on immunotherapeutic treatment, including in solid tumors (such as melanoma, lung tumors, lymphoma), given the recent success achieved, particularly using PD-L1 blockade to enhance anti-tumor immune responses [6,7].

To date, the prognostic and predictive implications of programmed death ligand-1 (PD-L1) in soft tissue tumors, mainly in liposarcomas, are not well known [8]. There is a constantly growing interest in studying the tumor microenvironment to improve therapeutic options in these tumors. The tumor microenvironment, mainly the immunohistochemical expression of CD3 and CD8 lymphocytes, was investigated in gastrointestinal stromal tumors and Ewing sarcoma, demonstrating that increased levels of these markers correlate with better survival and can be considered prognostic factors [9,10].

PD-L1 is a ligand of the PD-1 receptor; the latter belongs to the CD28 receptor family, with a role in the development of tumor anti-immunity, and is considered an attenuated mediator of the immune response by negatively regulating T-cell proliferation and function [11,12]. This ligand is expressed by immunocompetent cells such as B lymphocytes, T lymphocytes, macrophages, dendritic cells, and tumor cells, and its expression has been associated with poor prognosis in a variety of tumors [13,14,15,16,17].

Also, these effector cells, such as CD8+ cytotoxic T cells, upregulate the expression of the PD-1 receptor, blocking the interaction with PD-L1 ligands, resulting in the inhibition of tumor growth and progression, so research exploring the balance of the tumor microdomain and these negative signals generated by PD-L1 is needed [18,19,20].

Modern immunomodulatory therapeutic strategies have targeted the PD-1/PD-L1 axis, and different systems have been developed to evaluate and grade the immunohistochemical expression of PDL-1 in different tumor types, with the latter being considered the most reliable therapeutic predictive marker for response to immunotherapy [21,22]. Immunohistochemical expression of PD-L1 was evaluated in lung, gastrointestinal, breast, urothelial, and kidney tumors but also melanoma, head and neck, and hematologic cancers, using two scoring methods: the tumor proportion score (TPS) or tumor cell score (TC), representing the percentage of tumor cells with membranous positivity, irrespective of staining pattern and intensity, evaluated on at least 100 viable tumor cells, and the immune cell score (IC), representing the area occupied by PD-L1-positive immune cells relative to the entire tumor and peritumoral area. These evaluation scores are considered identical in practice and are usually classified into cut-off groups depending on the clone used and the tumor to be evaluated (e.g., for lung tumors, TPS < 1%, no PD-L1 expression, TPS 1–49%, low PD-L1 expression, TPS ≥ 50%, high PD-L1 expression or IC 0: < 1%, IC 1: 1–4%, IC 2: 5–9%, IC 3: >10%) [21,23,24].

Various studies report the presence of an intense CD8-positive cytotoxic T lymphocyte response in soft tissue tumors, especially those with high PD-L1 expression [25,26,27]. In this direction, we evaluated the expression of these cells using histochemical scoring assessment of CD8 (H-score) on liposarcomas.

In addition to PD-L1, a tumor suppressor protein, Maspin, has been shown to be a negative prognostic predictor marker in carcinomas [28,29] and soft tissue sarcomas [30,31,32]. We also aimed to study the expression of this marker in soft tissue tumors with adipose differentiation and to observe whether the expression of this marker was potentiated by PD-L1.

## 2. Materials and Methods

### 2.1. Case Selection

Forty-two consecutive liposarcomas cases diagnosed between 2016 and 2023 in the Department of Pathology of the Clinical County Emergency Hospital, Targu Mures, Romania, were included in the present study. Criteria of inclusion were as follows: patients who received a curative resection, without preoperative adjuvant therapy, with a diagnosis of liposarcoma, and a postoperative survival rate of ≥3 months. Other histological subtypes of sarcomas, epithelial or metastatic tumors, and cases from patients receiving palliative surgery were not included. Processing of the cases was approved by the Ethical Committee of the Clinical County Emergency Hospital, Targu Mures, Romania.

For all cases, the available slides with tumor cells were reanalyzed. We aimed to establish staging according to the most recent edition of the WHO Classification of Soft Tissue Tumors staging manual proposed in 2020 [5,33]. Tumors were also graded according to the FNCLCC grading system (Fédération Nationale des Centres de Lutte Contre le Cancer), defined by tumor differentiation, mitotic count, and necrosis.

### 2.2. Immunohistochemistry Analysis and Interpretation

In all cases, conventional slides were used for immunohistochemistry (IHC) assessment. After reviewing the hematoxylin and eosin-stained sections, two experienced pathologists chose one representative sample to be used for further IHC processing. For all 42 cases, we performed immunostaining for PD-L1 using two different monoclonal antibodies from Dako: PD-L1 IHC 22C3 pharmDx (DAKO North America, Inc, Santa Clara, United States) and PD-L1 IHC 28-8 pharmDX (DAKO North America, Inc, Santa Clara, United States). High pH retrieval was performed for the two antibodies. Both antibodies were ready to use (RTU). Immunostaining was performed automatically (for both antibodies) using the Autostainer Link 48 Dako.

After developing with diaminobenzidine (DAB) and counterstaining with hematoxylin, the TPS/TC was independently evaluated by two experienced pathologists, using cut-offs of less than 10% and higher or equal than 10%, for both antibodies, as well as the immunoreactivity of tumor cell and inflammation (tumor positivity with no inflammation = A, tumor negativity and inflammation = B, both tumoral and inflammation positivity = C, no tumoral and inflammation positivity = D). In cases in which the results differed between the two pathologists, the case was reevaluated by both pathologists and by the senior pathologist on the team. When necessary, immunostaining was performed on supplementary slides, for elucidation. To ensure the reproducibility of the immunohistochemical assessment, inter-observer agreement between the two independent pathologists was statistically evaluated using Cohen’s kappa coefficient (κ). The calculated value (κ = 0.82) indicated substantial concordance, confirming the consistency and reliability of the scoring results across all evaluated markers (PD-L1, CD4, CD8, CD20, CD68, and Maspin).

We also sought to evaluate the prevalence of CD8 High Score and Maspin in liposarcoma to correlate these markers with PD-L1 expressions. For Maspin, we used a system of quantification, which was previously published by our team and is based on the subcellular localization of this protease, and cases were classified as negative or with cytoplasm positivity with Maspin antibody BSB-92 [34,35,36]. The cytotoxic T lymphocytes in intra- and peritumoral regions were evaluated immunohistochemically using the CD8 antibody, Clone C8/144B Dako, establishing the H-Score using the QuantiScan quantification program of the Panoramic 250 Flash III device.

CD4 (clone 4B12 Dako), CD20 (clone L26 Dako), and CD68 (clone KP1 Dako) immunoreactivity was also assessed to explore the broader tumor immune microenvironment. These markers were recorded as absent (0) or present (1), based on unequivocal membranous or cytoplasmic staining in at least 5% of inflammatory cells, consistent with prior soft tissue sarcoma studies [36]. The binary approach was selected due to the low inflammatory background characteristic of liposarcoma and the exploratory nature of this analysis.

### 2.3. Statistical Analysis

The results were further analyzed using GraphPad Prism 10 (software-free version). For correlations between the clinicopathological features and different patterns of IHC staining for PD-L1, CD8, and Maspin, chi-squared and Fisher’s exact test were used. For all analyses, a *p*-value under 0.05 and a 95% confidence interval were considered statistically significant. Associations between CD4, CD20, CD68 status, and PD-L1 and CD8 expression were analyzed using Fisher’s exact test. A *p*-value < 0.05 was considered statistically significant. Overall survival (OS) was calculated from the date of diagnosis to death or last follow-up. Survival status was coded as 0 = deceased and 1 = alive. Kaplan–Meier survival curves were generated, and differences between groups were assessed using the log-rank test. Patients were stratified according to PD-L1 expression (TPS and TC < 10% vs. ≥ 10%) and CD8 immunoscore (H-score < 2 vs. ≥2). Due to the retrospective nature, heterogeneous histologic subtypes, and limited number of events, these analyses were considered exploratory.

## 3. Results

### 3.1. Clinicopathological Characteristics of the Study Cohort

**Figure 1:**
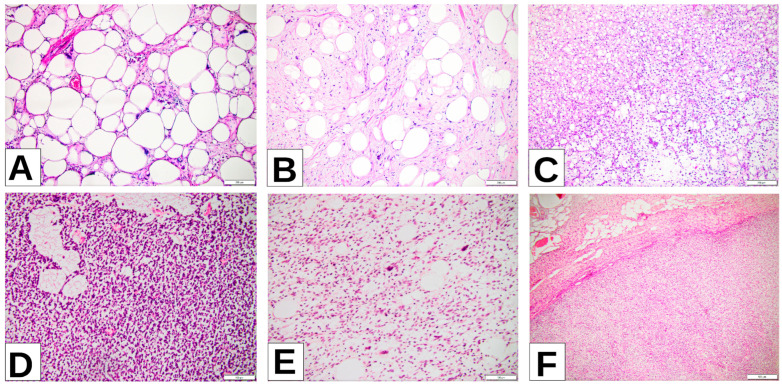
(**A**) WDLPS lipoma-like (well-differentiated liposarcoma lipoma-like) images of the right thigh: sheets of well-differentiated adipocytes with only nuclear atypia. (**B**) ALT (atypical lipomatous tumor) of the neck: atypical stromal cells predominate, with scattered well-differentiated adipocytes. (**C**) MLPS (myxoid liposarcoma), left thigh: signet ring lipoblasts among primitive nonlipogenic mesenchymal cells arranged in a prominent myxoid stroma. (**D**) RCL (round-cell liposarcoma), retroperitoneum: hypercellular area with round cells among thin compressed capillaries in between tumoral cells. (**E**) PLPS (pleomorphic liposarcoma), retroperitoneum: high-grade sarcoma with spindle cell morphology and variable number of pleomorphic lipoblasts. (**F**) DDLPS (dedifferentiated liposarcoma), retroperitoneum: abrupt transition from well-differentiated component in the upper half of the image to the dedifferentiated nonlipogenic component in the lower half of the picture.

In total, 42 patients with liposarcomas were diagnosed between the age of 37 and 81 years, with predominance of the male gender. Most of the tumors were in the retroperitoneum and extremities, with less than 30% located on the trunk and head and neck. Based on grading, almost 50% of tumors were well differentiated (G1), followed by poorly differentiated tumors (G3). A third of the cases were diagnosed in advanced stages (pT4), followed by the pT2 stage and pT3 stage, with only 15% diagnosed at an early stage (pT1) (Table 1, Figure 1).

### 3.2. PD-L1 Expression Using DAKO 22C3 and 28-8 Clones

**Figure 2:**
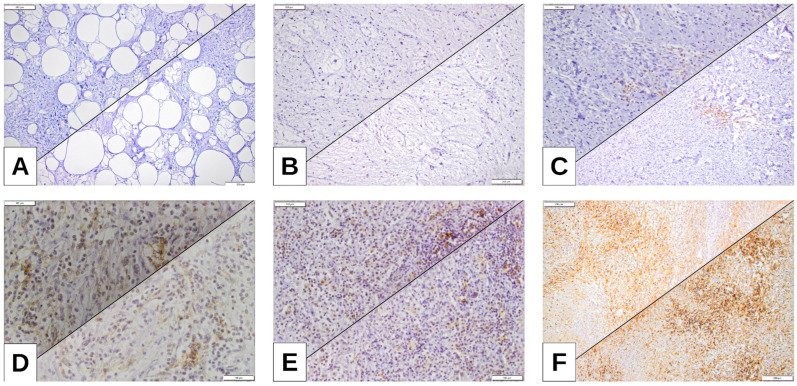
(**A**) ALT (atypical lipomatous tumor) of the neck, tumor proportion score (TPS) 0%; tumor cell score (TC) 0%. (**B**) MLPS, left thigh, TPS 1%, TC1%. (**C**) PLPS, inguinal area, TPS 5%, TC 8%. (**D**) DDLPS, retroperitoneum, TPS 10%, TC 12.5%. (**E**) DDLPS, retroperitoneum TPS 15%, TC 15%. (**F**) DDLPS, retroperitoneum, highest expression of TPS (PDL-1 22C3) and TC (PDL-1 28-8), TPS 32.5%, TC 35%.

Most of the cases evaluated with the two PD-L1 clones showed a low tumor proportion score or tumor cell score (low TPS/low TC), with higher expression (high TPS/high TC) observed in 9.5% of cases (n = 4) for clone 22C3 and 11.9% of cases (n = 5) for clone 28-8 (Table 2, Figure 2).

### 3.3. Correlation Between PD-L1 Expression and CD8+ Tumor-Infiltrating Lymphocytes

**Figure 3:**
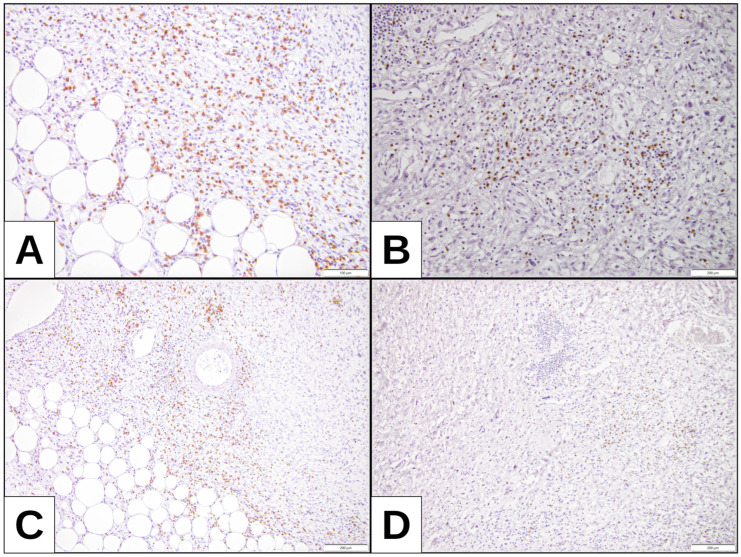
CD8 expression: (**A**,**C**) DDLPS with higher expression of TPS and TC (32.5% and 35%, respectively) and CD8 positivity, H-score assessment 19.88. (**B**,**D**) DDLPS with no TPS and TC expression and CD8 positivity, with H-score assessment 17.28.

To explore the immune microenvironment, CD8+ T-cell density was quantified using the H-score method. A significant correlation was found between higher PD-L1 expression and increased CD8+ infiltration (*p* = 0.007 for 22C3; *p* = 0.0331 for 28-8). Over 85% of cases had low CD8+ infiltration (H-score < 10%), whereas high H-scores were identified only in tumors with elevated PD-L1 expression (Table 3, Figure 3).

This finding suggests that increased PD-L1 expression is associated with an adaptive immune response characterized by cytotoxic T-cell infiltration.

Out of a total of 42 patients, at least 85% of cases presented an H-score of less than 10%, indicating a low expression of TPS/TC, regardless of the clone used. In both the 22C3 and 28-8 clones, only two cases had an H-score of over 10% and high TPS/TC score. In the remaining cases, the 28-8 clone recorded more instances of high TC along with an H-score above 10% (Table 3, Figure 3).

### 3.4. PD-L1 Expression in Tumor and Inflammatory Cells

We next examined PD-L1 immunoreactivity in both tumor and inflammatory compartments. For both clones, at least 85% of cases did not show tumoral or inflammatory positivity (D). In cases without tumoral and inflammatory positivity, only one case was identified with both clones (B) with an H-score of more than 10%. For inflammation and tumoral positivity (C), two cases were identified with an H-score of less than 10%, while for an H-score of more than 10%, only one case was identified with the 28-8 clone and two cases for 22C3. In cases with tumoral positivity without inflammation, there were two positive cases for the 28-8 clone (A) (Table 4).

### 3.5. Association of PD-L1 Expression with Tumor Differentiation

To better understand the relationship between PD-L1 expression and the histopathological aggressiveness of liposarcomas, we next evaluated its association with tumor differentiation grade according to the FNCLCC grading system.

The majority of the analyzed liposarcomas were well-differentiated tumors (G1), with a TPS/TC score below 10%; only one isolated case was diagnosed with TC greater than 10% for the 28-8 clone. Regarding advanced stages of tumor differentiation, an identical number of tumors were found in both clones (N = 10 for G2 and N = 14 for G3). For the 22C3 clone, tumors with TPS over 10% were identified only in aggressive tumors (G3). In the case of the 28-8 clone, a similar number of cases with TC over 10% were identified, but one case was also found at a well-differentiated stage (G1) (Table 5).

### 3.6. Correlation Between PD-L1 and Maspin Expression

Given the potential interaction between immune checkpoint activation and tumor suppressor pathways, we further investigated the relationship between PD-L1 expression and Maspin positivity across liposarcoma cases.

**Figure 4:**
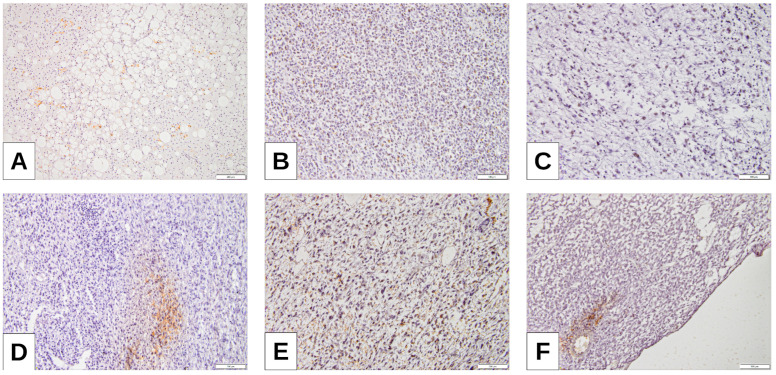
Maspin expression: (**A**) WDLPS low positivity of Maspin; (**B**) MLPS with hypercellular component with Maspin positivity within tumoral cells; (**C**,**D**) DDLPS with higher expression of TPS and TC (32.5% and 35%, respectively), also showing Maspin positivity; (**E**,**F**) PLPS and RCL with higher Maspin positivity.

Most cases were Maspin-negative and showed low expression in both TPS/TC clones (Figure 4). For both PD-L1 clones, 7 Maspin-positive cases were identified, with equal distribution of the percentage of tumor expression between clones (Table 6).

### 3.7. Exploratory Immune Profiling

**Figure 5:**
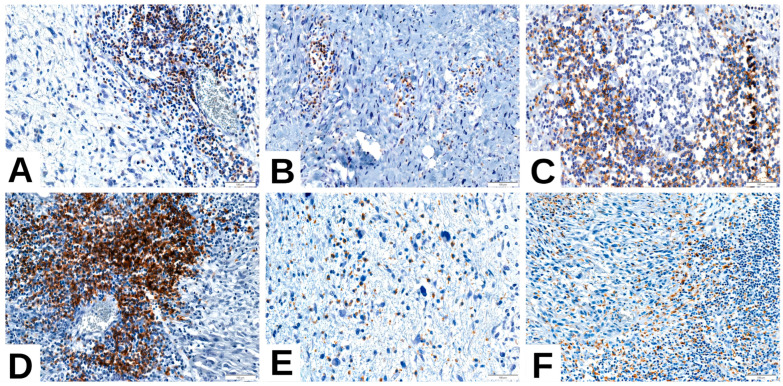
Immune profile of liposarcoma—representative features. (**A**,**B**) CD4-positive T-cell infiltrate in (**A**) dedifferentiated liposarcoma (DDLPS) and (**B**) pleomorphic liposarcoma (PLPS); (**C**,**D**) CD20-positive B-cell aggregates in DDLPS; (**E**) CD68-positive macrophages in myxoid liposarcoma (MLPS); (**F**) CD68-positive macrophages in DDLPS.

As an exploratory extension, we evaluated immune cell markers (CD4, CD20, CD68) in the same cohort of 42 cases (Figure 5). CD20 and CD68 positivity were significantly enriched in PD-L1-high tumors and in CD8-high cases (Table 7). CD4 positivity was rare but showed a trend toward association with PD-L1-high tumors (Table 7). These findings indicate a minority immune-inflamed subset of tumors characterized by coordinated CD8+, CD20+, and CD68+ infiltration.

### 3.8. Survival Analysis

The median follow-up was 32.5 months (range 1–120 months). A total of 21/42 patients (50%) died during follow-up, enabling estimation of the median overall survival (OS), which was 48.0 months.

Kaplan–Meier curves showed no statistically significant differences in OS between PD-L1-high (≥10%) and PD-L1-low cases or between CD8-high (H-score ≥ 2) and CD8-low tumors (log-rank *p* > 0.05). Although limited by sample size, the curves suggested a trend toward shorter OS in immune-inflamed tumors, consistent with the more aggressive histological subtypes of liposarcoma.

## 4. Discussion

### 4.1. PD-L1 Expression and Its Clinical Significance in Sarcomas

To date, The Food and Drug Administration (FDA) has approved four PD-L1 IHC assays as companion diagnostics (CDx) for guiding immunotherapy treatments. These include Dako 22C3 for pembrolizumab in various solid tumors, Ventana SP142 for atezolizumab in urothelial carcinoma and NSCLC, Dako 28-8 for a combination of ipilimumab and nivolumab in NSCLC, and Ventana SP263 for atezolizumab in NSCLC. These assays help determine which patients will benefit most from specific PD-L1-targeted therapies based on tumor biomarker profiles [37]. A comparative study published by Maule et al. evaluated the immunohistochemical expression of PD-L1 on 418 lung tumors using three clones from different manufacturers (Dako 22C3, Dako 28-8, and Ventana SP142). Their study revealed that 22C3 was positive in 94.2% of tumor cases, while 28-8 and SP142 were positive in 77.0% and 28.8% of cases, respectively. Among NSCLC specimens, 22C3 had the highest positivity rate at 95.2%. The study also found that when tests disagreed on PD-L1 status, 22C3 was often the test that returned a positive result. Specifically, 62.3% of cases with differing PD-L1 status were 22C3+/28-8+/SP142-, suggesting the higher sensitivity of 22C3 in clinical practice [38]. In our liposarcoma study, using the two different Dako clones (22C3, 28-8), no statistically significant difference in specificity between the two clones was identified.

The immune checkpoint protein PD-L1 has gained significant attention in recent years due to its role in inhibiting T cell activation and proliferation by binding to PD-1 on T cells, thereby allowing tumor cells to evade the host immune surveillance system. This mechanism of immune evasion has made PD-L1 a critical target in cancer immunotherapy, particularly in the context of immune checkpoint inhibitors. These inhibitors have demonstrated notable success in treating different types of cancer, providing new approaches for personalized treatment [39]. However, despite the success of immune checkpoint blockade in some cancers, the efficacy of these therapies in certain cancers, such as bone and soft tissue sarcomas, remains less promising [40].

Wang et al. published a meta-analysis in 2020 that included 39 independent studies enrolling a total of 3680 patients to investigate the prognostic significance of PD-L1 expression in sarcomas. The results showed a consistent association between increased PD-L1 expression and poor patient outcomes. Specifically, PD-L1 overexpression was found to be a significant predictor of poorer overall survival, metastasis-free survival, and event-free survival. In addition to the survival outcomes, this meta-analysis also demonstrated that high PD-L1 expression in sarcomas was associated with an increased rate of tumor metastasis and more advanced tumor differentiation grading [23]. Also in our study, poorly differentiated tumors (G3) were associated with increased expression of PD-L1 TPS/TC, for both types of clones used, compared to well-differentiated tumors, which showed low levels of TPS/TC, except for one case of an atypical lipomatous tumor (G1) in which increased expression of PD-L1 TC was identified in the PD-L1 Dako 28-8 clone.

### 4.2. CD8+ Tumor-Infiltrating Lymphocytes and the PD-L1/PD-1 Axis

Recent studies have shown that the tumor microenvironment of sarcomas is highly heterogeneous, and immune infiltration varies significantly between subtypes, impacting immunotherapy response and overall survival (OS). CD8+ immune cells are the most crucial for anti-tumor response, although they are found in much lower numbers in liposarcomas compared to other tumors such as gastrointestinal stromal tumors, myxofibrosarcomas, and pleomorphic sarcomas. However, among liposarcomas, dedifferentiated liposarcomas (DDLPSs) showed the highest number of CD8+ tumor-infiltrating lymphocytes (TILs), which are also associated with improved OS, according to the study by Dancsok et al. [41]. Our study also confirmed a lack of CD8+ tumor-infiltrating lymphocyte (TIL) response in most cases. Using an H-score to quantify CD8 population, less than 8% of the total cases examined showed infiltration greater than 10%, for both clones.

The same previously mentioned meta-analysis reveals a significant correlation between elevated PD-L1 expression and an increased presence of tumor-infiltrating lymphocytes (TILs), in alignment with findings from previous studies on sarcomas and other cancers. This association suggests that sarcomas with increased PD-L1 expression may have enhanced the mechanisms of immune evasion, potentially influencing tumor progression and response to immune therapies. A more detailed examination of T cell subtypes revealed that PD-L1 overexpression was predominantly linked with PD-1+ T cells, CD3+ T cells, and CD8+ T cells, but interestingly not with CD4+ T cells [42,43,44]. In our liposarcoma study, a statistically significant correlation between PD-L1 TPS/TC expression and CD8 H-Score assessment was also found, independent of the analyzed clone. Thus, low PD-L1 TPS/TC expressions were associated with reduced expression of CD8 lymphocytes quantified by H-score assessment (<10%), and increased levels of intratumoral PD-L1 expression were associated with increased CD8 H-score (>10%), respectively, for both clones used. This specificity of T cell subtype interaction underscores the complex role of the PD-1/PD-L1 axis in tumor immune tolerance. The PD-1 receptor, expressed on various T cells, including CD8+ T cells, plays a critical role in dampening the immune response against tumor cells. PD-L1 on tumor cells binds to PD-1 on T cells, thereby inhibiting the cytotoxic activity of CD8+ T cells and possibly other immune cells, including CD4+ T cells [45,46,47]. This inhibition contributes to the tumor’s ability to escape immune detection and destruction, allowing cancer to persist and potentially progress. Recent investigations have further emphasized the biological interplay between PD-L1 expression and CD8+ tumor-infiltrating lymphocytes in liposarcomas and other soft tissue sarcomas, underlining their prognostic and therapeutic significance. In particular, Torres et al. demonstrated that dedifferentiated liposarcomas with higher CD8+ T-cell densities exhibited a more favorable response to immune checkpoint blockade [48]. Additionally, Huang et al. and Roulleaux Dugage et al. highlighted the influence of the tumor immune microenvironment on immunotherapy efficacy, while Italiano et al. reported modest clinical outcomes in PD-1/PD-L1–targeted trials, with variable correlation with biomarker expression [49,50,51]. These recent findings reinforce our current observations and support the growing evidence that PD-L1 overexpression and CD8+ infiltration represent interdependent features of an immune-evasive tumor phenotype in liposarcomas. However, despite this apparent link between high PD-L1 expression and increased TILs, clinical efforts targeting the PD-1/PD-L1 axis in sarcomas have not yielded satisfactory results [52,53]. This lack of efficacy highlights the complexity of immune interactions in sarcomas, where PD-L1 expression may not be the sole determinant of immune evasion. Further research is needed to elucidate the specific role of PD-L1 in soft tissue tumors, even liposarcomas immune evasion, and to explore alternative strategies to enhance the effectiveness of immunotherapy in this type of cancer. Understanding the underlying mechanisms of PD-L1 expression and its interaction with TILs will be crucial in developing more effective treatment regimens for sarcoma patients.

In a sarcoma study published by D’Angelo et al., tumor expression of PD-L1 was observed in 6 (12%) of cases, while immune cell expression was more evident in these specimens: 15 (30%) of lymphocytes and 29 (58%) of macrophages showed PD-L1 positivity. Among the sarcoma subtypes, gastrointestinal stromal tumors (GISTs) had the highest rate of PD-L1 expression at 29% (4/14 samples). In addition, PD-L1 expression was also observed in two other subtypes of poorly differentiated sarcomas, (G3) radiation-associated pleomorphic sarcoma and a spindle cell sarcoma; in the latter, the percentage of PD-L1 expression in immune cells was high. These findings suggest that, although PD-L1 expression in tumor cells is relatively low, the immune microenvironment plays a significant role in modulating immune checkpoint activity in sarcomas [9]. In our liposarcoma study, it was observed in both clone 22-8 and clone 22C3 that the percentage of PD-L1 expression in inflammatory cells was increased in cases with high PD-L1 TPS/TC expression (cut-off ≥ 10) and consequently with a CD8 H-score ≥ 10.

While the present study focused on CD8+ cytotoxic T lymphocytes as the principal effector subset mediating anti-tumor immunity, we acknowledge that the immune microenvironment of liposarcomas is multifaceted. Other cell populations, including CD4+ helper T cells, B-lymphocytes, and tumor-associated macrophages, also contribute to the modulation of immune responses and may influence PD-L1 expression patterns [41,54]. However, as our methodological design specifically targeted the CD8+ compartment and its association with PD-L1 and Maspin, broader immune profiling was beyond the intended scope of this work. Future studies incorporating multiplex immunohistochemistry could provide a more comprehensive understanding of the immune ecosystem in liposarcomas. Beyond CD8+ cytotoxic T-cell evaluation, we also explored the distribution of CD4, CD20, and CD68 immune cell subsets. Although most liposarcomas lacked substantial immune infiltration, CD20 and CD68 positivity clustered within PD-L1-high and CD8-rich **cases**, supporting the presence of a limited but biologically relevant immune-inflamed phenotype. This observation aligns with recent data in soft tissue sarcomas, where B-cell- and macrophage-rich tertiary lymphoid structures correlate with responses to immune checkpoint therapy. The predominance of immune-cold tumors and the existence of a smaller immune-inflamed subset may partly explain the heterogeneous clinical performance of immunotherapy in liposarcoma [41,54,55].

### 4.3. Biological Interplay Between Maspin and PD-L1 in Liposarcomas

Despite numerous studies suggesting that Maspin may influence tumor behavior and patient outcomes in breast, prostate, gastric, and colorectal cancers, there is incomplete data regarding its expression and prognostic implications in STS. In this regard, there are three reports describing Maspin expression in soft tissue sarcomas, one of them investigated the association between cytoplasmic expression of Maspin and prognosis of STS patients. Specifically, cytoplasmic expression of Maspin was strongly correlated with higher FNCLCC grade, presence of distant metastasis, and significantly shorter disease-free survival (DFS) and overall survival (OS). These findings suggest that Maspin may contribute to the aggressive nature of STS by influencing tumor differentiation and metastatic potential [31,32,33]. In our liposarcoma study, Maspin-negative cases were associated with low PD-L1 TPS/TC expressions, and those with high PD-L1 TPS/TC expression showed cytoplasmic Maspin positivity. Moreover, in our study, almost all cases with high PD-L1 TPS/TC expression are poorly differentiated tumors (G3 FNCLCC), with a statistically significant association between increased TPS/TC levels and Maspin positivity for both clones studied. The observed statistical correlation between Maspin and PD-L1 expression in our cohort may also have a biological explanation. Maspin, a member of the serine protease inhibitor family (serpin B5), has been shown to exert tumor-suppressive effects in certain epithelial malignancies, but its cytoplasmic localization can paradoxically promote tumor progression and immune tolerance. Several recent studies suggest that cytoplasmic Maspin may activate NF-κB-dependent transcription and facilitate epithelial–mesenchymal transition (EMT), thereby indirectly enhancing PD-L1 expression and immune checkpoint activation. Moreover, the NF-κB and PI3K/AKT signaling pathways, both linked to Maspin’s non-canonical activity, are known to drive PD-L1 upregulation and contribute to the formation of an immunosuppressive tumor microenvironment. These molecular interactions may provide a biological rationale for the concomitant expression of Maspin and PD-L1 observed in our liposarcoma cases [56,57,58,59].

### 4.4. Prognostic and Predictive Value of PD-L1 as a Biomarker

The relationship between PD-1/PD-L1 expression and prognosis has been widely explored in various cancers, including sarcoma. Prior studies have highlighted the role of PD-1 and PD-L1 as critical immune checkpoint regulators that influence tumor progression and patient survival. In gastric and lung cancers, PD-L1 expressions were detected in approximately half of the cases, where it was identified as an independent prognostic factor for overall survival (OS). In these malignancies, PD-L1 expressions were found to be a significant negative predictor of OS, age, tumor size, histology, location, surgical outcome, and response to adjuvant treatment [60,61]. These findings strongly suggest that PD-L1 plays a crucial role in the progression of various solid tumors and serves as a potential biomarker for patient prognosis. In addition to its prognostic significance in various cancers, PD-L1 expression is increasingly being recognized as a crucial biomarker for predicting the effectiveness of PD-1/PD-L1 blockade therapies. The role of PD-L1 as a predictor of treatment response has been explored in several clinical trials, underscoring its potential to guide therapeutic decisions [62,63]. While PD-L1 expression holds potential as a biomarker for predicting the efficacy of PD-1/PD-L1 inhibitors, one of the major challenges in utilizing PD-L1 as a clinical tool is the lack of standardized criteria for its assessment. Variability in the definitions of positive PD-L1 expression has led to inconsistent results across different clinical trials. This inconsistency can complicate the interpretation of PD-L1 as a reliable predictive marker for treatment response. Immunohistochemistry (IHC) is widely used for PD-L1 detection, with many studies advocating for its use. However, there is significant variability in the antibodies and cut-off values applied for defining positive PD-L1 expression, which complicates the interpretation of results [64,65]. These findings suggest that the selected cut-off value may significantly influence the prognostic utility of PD-L1 expression, emphasizing the need for standardized criteria to ensure consistency in clinical practice. This variability in cut-off values is a notable concern, as it may affect the comparability of results across studies and impact the ability to draw definitive conclusions about the prognostic value of PD-L1. While PD-L1 expression remains a promising biomarker for predicting outcomes and treatment responses, significant challenges remain regarding the standardization of assessment methods and cut-off values. Further studies are necessary to establish consistent and reliable protocols for PD-L1 testing, incorporating advanced techniques and comprehensive analyses to refine its prognostic and predictive capabilities in clinical practice, particularly for patients with sarcomas.

### 4.5. Limitations of the Study

Although the present study provides novel insights into the relationship between PD-L1, CD8+ cytotoxic T cells, and Maspin expression in liposarcomas, several aspects should be considered when interpreting the results. The number of analyzed cases was relatively limited, reflecting the overall rarity and histological heterogeneity of these tumors. Nevertheless, the inclusion of multiple liposarcoma subtypes allows for a broader overview of immune variability within this group.

In addition, while we expanded the immune evaluation beyond CD8 to include CD4, CD20, and CD68, these markers were assessed in an exploratory, binary manner rather than via quantitative spatial or multiplex immunophenotyping. This approach enabled consistent interpretation across cases but did not fully capture the complexity of the tumor immune microenvironment or tertiary lymphoid architecture.

Importantly, this work represents an initial step toward characterizing the immuno-oncologic profile of liposarcomas using standardized PD-L1 assays. The findings support the existence of predominantly immune-cold tumors with a minority immune-inflamed subset and establish a foundation for future prospective, multicenter studies with larger cohorts. Such studies may incorporate multiplex panels, digital image analysis, spatial immune profiling, and RNA-based immune signatures to further refine the immunological landscape of liposarcomas and identify predictive biomarkers for immunotherapy.

Consistent with prior soft tissue sarcoma studies, survival analysis did not show statistically significant differences according to PD-L1 or CD8 status, likely reflecting the relatively small cohort size, histologic diversity, and limited number of events. Nonetheless, the observed trend toward shorter OS in PD-L1-positive and CD8-high tumors supports the concept that a small immunologically active subset of liposarcomas may exhibit more aggressive clinical behavior and potential sensitivity to immune modulation.

## 5. Conclusions

PD-L1 expression has become an important focus in cancer immunotherapy, particularly as a biomarker for predicting response to PD-1/PD-L1 inhibitors. The FDA has approved multiple PD-L1 IHC assays for various cancers, helping to guide treatment decisions. However, variability in test results and a lack of standardization in PD-L1 assessment methods continue to pose challenges. In sarcomas, including liposarcomas, while high PD-L1 expression is linked with poor prognosis and increased immune evasion, clinical trials targeting PD-1/PD-L1 have had limited success. Despite these challenges, understanding the complex interactions between PD-L1, tumor-infiltrating lymphocytes (TILs), and immune evasion mechanisms remains crucial in improving immunotherapy strategies. Additionally, further research is needed to establish standardized criteria for PD-L1 testing and explore alternative therapeutic strategies to enhance the effectiveness of immune checkpoint inhibitors in soft tissue tumors. In addition to widespread immune-cold features, our exploratory analysis identified a small subset of liposarcomas with concurrent PD-L1 expression, CD8+ infiltration, and CD68/CD20 positivity, suggesting an immune-inflamed niche that may represent the fraction most likely to benefit from immunotherapy. Exploratory Kaplan–Meier analysis did not reveal statistically significant survival differences by PD-L1 or CD8 status, underlining the need for larger prospective cohorts with longer follow-up periods.

## Figures and Tables

**Table 1:** Clinicopathological parameters of the included cases.

Parameter Median Age (Years)		Values (n = 42) 61 ± 1.82 (%)
Gender	Male Female	25 (59.5) 17 (40.5)
Tumor location	Head and neck Trunk Retroperitoneum Extremities	4 (9.5) 7 (16.5) 16 (38) 15 (36)
Histological subtype and grade of differentiation (G)	ALT/WDLP (G1) Myxoid LPS (G2) Pleomorphic/DDLPS/poorly differentiated LPS (G3)	18 (42.8) 10 (23.8) 14 (33.4)
Depth of invasion (pT stage)	pT1 pT2 pT3 pT4	6 (15.2) 13 (30.1) 9 (21.4) 14 (33.3)

**Table 2:** PD-L1 tumor proportion score (TPS) and tumor cell score (TC) expression using two different DAKO clones (see explanation in text—Section 4).

TPS	PD-L1 DAKO 22C3	TC	PD-L1 DAKO 28-8
<10%	38 (90.5)	<10%	37 (88.1)
≥10%	4 (9.5)	≥10%	5 (11.9)

**Table 3:** Correlation between immunohistochemical quantification of tumor proportion score (TPS)/tumor cell score (TC) regarding PD-L1 expression using two different clones and H-score.

Clone	TPS/TC	H-Score < 10% (%)	H-Score > 10% (%)	*p* Value
22C3 (N = 42)	Low TPS	38 (90)	0 (0)	***p* = 0.007**
	High TPS	2 (5)	2 (5)	
28-8 (N = 42)	Low TC	36 (85)	1 (2.5)	***p* = 0.0331**
	High TC	3 (7.5)	2 (2.5)	

The bold of *p* value below 0.05 means that the value is statistically significant.

**Table 4:** Correlation between PD-L1 immunoreactivity of tumor cell and inflammation (T/INFL) using the two different Dako clones and H-score. T/INFL: tumor positivity with no inflammation = A, tumor negativity and inflammation = B, both tumoral and inflammation positivity = C, no tumoral and inflammation positivity = D.

PD-L1 Clone	H-Score	T/INFL				*p* Value
		A (%)	B (%)	C (%)	D (%)	
28-8	<10%	2 (5)	0	2 (5)	36 (85)	***p* = 0.0105**
	≥10%	0	1 (2.5)	1 (2.5)	0	
22C3	<10%	0	0	2 (5)	37 (87.5)	***p* = 0.0009**
	≥10%	0	1 (2.5)	2 (5)	0	

The bold of *p* value below 0.05 means that the value is statistically significant.

**Table 5:** PD-L1 TPS/TC expression correlation with tumor differentiation grades.

PD-L1 TPS/TC		Tumor Differentiation Grade			*p* Value
		G1 (%)	G2 (%)	G3 (%)	
22C3	<10%	18	10	11	***p* = 0.0422**
	≥10%	0	0	3	
28-8	<10%	17	10	11	*p* = 0.2
	≥10%	1	0	3	

The bold of *p* value below 0.05 means that the value is statistically significant.

**Table 6:** PD-L1 TPS/TC expression correlation with Maspin.

PD-L1 TPS/TC		Maspin		*p* Value
		Positive	Negative	
22C3	<10%	4	35	***p* = 0.003**
	≥10%	3	0	
28-8	<10%	4	34	***p* = 0.0113**
	≥10%	3	1	

The bold of *p* value below 0.05 means that the value is statistically significant.

**Table 7:** Exploratory correlations between immune markers and PD-L1/CD8 status (n = 42).

Marker	Category	PD-L1 < 10%	PD-L1 ≥ 10%	*p*-Value	CD8 Low	CD8 High	*p*-Value
CD4	Negative	35	2	**0.022**	30	7	0.12
	Positive	1	2		1	2	
CD20	Negative	33	0	**0.009**	30	3	**0.0085**
	Positive	3	4		1	3	
CD68	Negative	32	0	**0.00076**	28	4	**0.0079**
	Positive	4	4		3	5	

The bold of *p* value below 0.05 means that the value is statistically significant.

## Data Availability

The original contributions presented in this study are included in the article. Further inquiries can be directed to the corresponding author.

## Abbreviations

The following abbreviations are used in this manuscript:

ALT	Atypical lipomatous tumor
WDLS	Well-differentiated liposarcoma
DDLPS	Dedifferentiated liposarcoma
MLS	Myxoid liposarcoma
PLPS	Pleomorphic liposarcoma
MPLS	Myxoid pleomorphic liposarcoma
PD-1	Programmed cell death receptor-1
PD-L1	Programmed death ligand 1
IHC	Immunohistochemistry
IC	Immune cell score
H-score	Histochemical score
TPS	Tumor proportion score
TC	Tumor cell score
TIL	Tumor-infiltrating lymphocyte
FNCLCC	Fédération Nationale des Centres de Lutte Contre le Cancer
